# Supplementary material for: Targeting Pseudomonas aeruginosa biofilm with an evolutionary trained bacteriophage cocktail exploiting phage resistance trade-offs
Source: Nat Commun. 2024 Oct 3;15:8572. doi: 10.1038/s41467-024-52595-w (PMC11450229; doi:10.1038/s41467-024-52595-w)
Supplement: Supplementary file 1 — Supplementary Information [file 41467_2024_52595_MOESM1_ESM.pdf]

Supplementary Information

Targeting *Pseudomonas aeruginosa* biofilm with an evolutionary trained bacteriophage cocktail exploiting phage resistance trade-offs

Fabian Kunisch[1,2,3,4], Claudia Campobasso[5,6], Jeroen Wagemans[5], Selma Yildirim[7], Benjamin K Chan[3,4], Christoph Schaudinn[8], Rob Lavigne[5], Paul E Turner[3,4,9], Michael J Raschke[1,10], Andrej Trampuz[2,7*], Mercedes Gonzalez Moreno[2,7]

[1]Faculty of Medicine, Universität Münster, Münster, Germany
[2]Center for Musculoskeletal Surgery, Charité – Universitätsmedizin Berlin, Corporate Member of Freie Universität Berlin and Humboldt-Universität zu Berlin, Berlin, Germany
[3]Department of Ecology and Evolutionary Biology, Yale University, New Haven, USA
[4]Center for Phage Biology and Therapy, Yale University, New Haven, USA
[5]Department of Biosystems, KU Leuven, Leuven, Belgium
[6]Department of Biology, Università di Pisa, Pisa, Italy
[7]Berlin Institute of Health at Charité – Universitätsmedizin Berlin, BIH Center for Regenerative Therapies (BCRT), Berlin, Germany
[8]Advanced Light and Electron Microscopy (Zentrum für Biologische Gefahren und Spezielle Pathogene 4), Robert Koch Institute, Berlin, Germany
[9]Program in Microbiology, Yale School of Medicine, New Haven, USA
[10]Department of Trauma, Hand and Reconstructive Surgery, Universitätsklinikum Münster, Münster, Germany

*Correspondence: Andrej Trampuz, andrej.trampuz@qut.edu.au

Figures

Figure S1: Overview of the bacteriophages and bacterial strains included in the evolution assay.

Figure S2: Bacteriophage activity analysis during the evolution assay.

Figure S3: Overview of the isolation of evolved bacteriophages after rounds 15 and 30.

Figure S4: Overview of the tertiary structure for FJK_gp62 and TTPA.

Figure S5: Characterisation of phage treated Paer09 mutants.

a

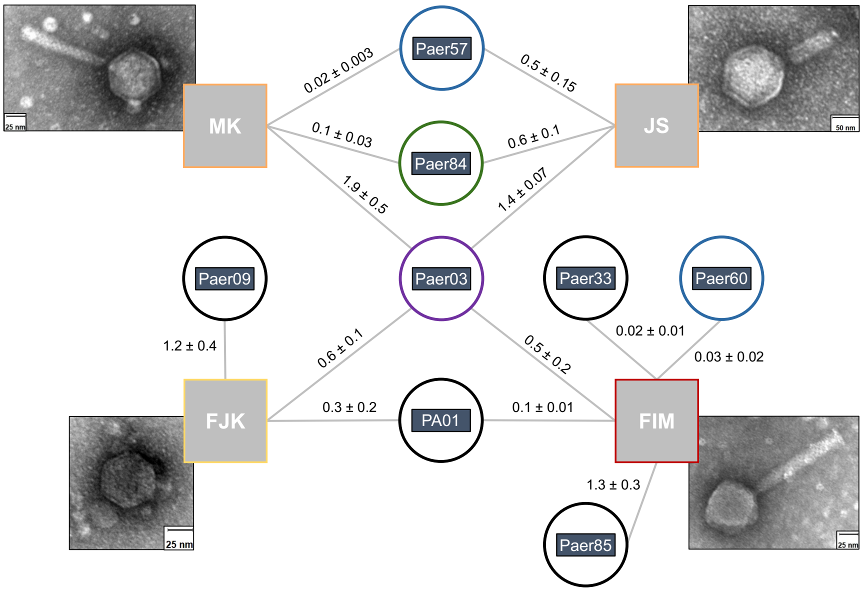
MK
JS
FJK
FIM
Paer57
Paer84
Paer03
Paer09
Paer33
Paer60
PA01
Paer85
0.02 ± 0.003
0.5 ± 0.15
0.1 ± 0.03
0.6 ± 0.1
1.9 ± 0.5
1.4 ± 0.07
1.2 ± 0.4
0.6 ± 0.1
0.5 ± 0.2
0.02 ± 0.01
0.03 ± 0.02
0.3 ± 0.2
0.1 ± 0.01
1.3 ± 0.3
25 nm
50 nm
25 nm
25 nm

b

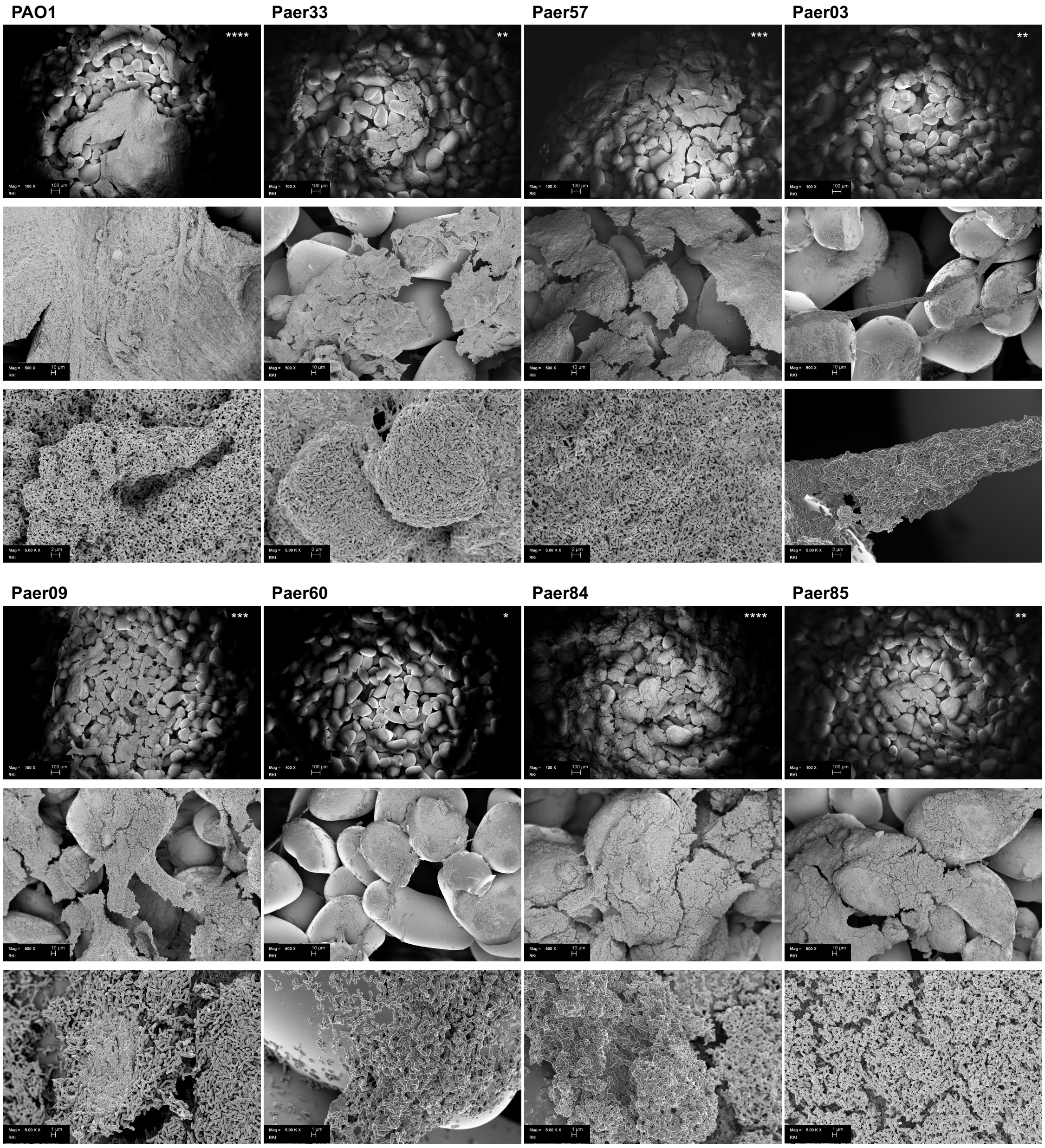
PAO1
Paer33
Paer57
Paer03
****
**
***
**
Paer09
Paer60
Paer84
Paer85
***
*
****
**

Fig. S1: Overview of the bacteriophages and bacterial strains included in the evolution assay.

a Transmission electron microscopy images of the four ancestral bacteriophages (*Pakpunavirus*: MK/JS, orange square; *Bruynoghevirus*: FJK, yellow square and *Pbunavirus*: FIM, red square) and their host range (connecting lines; numbers indicate EOP) among the eight *P. aeruginosa* strains included in the *in vitro* evolution and belonging to four genomic clusters (Cluster 1: PAO1/Paer09/Paer33/Paer85, black circle; Cluster 2: Paer57/Paer60, blue circle; Cluster 3: Paer03, violet circle; Cluster 4: Paer84, green circle). The efficacy of plating (EOP) is shown as mean with standard error of the mean of four replicates, except for phage JS and MK (Paer57), for which there are duplicates. **b** Scanning electron microscopy images of the pre-stablished 24-h-biofilm of each *P. aeruginosa* strain included in the evolution assay displayed at three different magnifications (top row – Mag 100 X; middle row – Mag 500 X; bottom row – Mag 5000 X). The number of stars in the top row indicate the biofilm density (more stars corresponding to higher biofilm density) for each strain in comparison to one another.

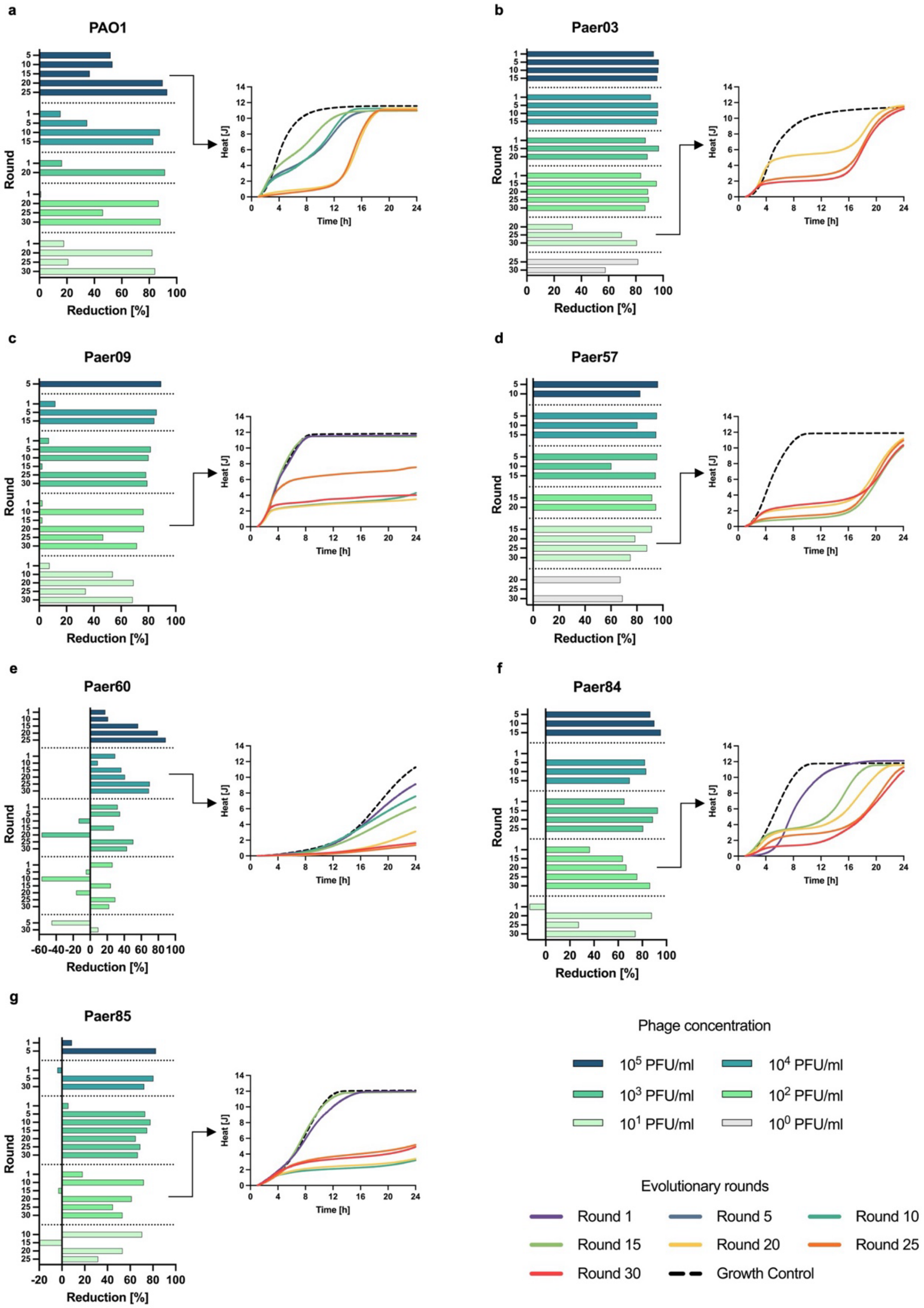

Fig. S2: Bacteriophage activity analysis during the evolution assay.

a Heat reduction (%) plot of PAO1 exposed to the phage mixture at different concentrations (PFU/ml) – determined ex post – compared to the growth control sample in different rounds of the evolution after 8 h of monitoring. Calorimetric heat (J) curves measured during a 24-h exposure of PAO1 to phages at the same concentration within different evolutionary rounds. **b** Identical setup for the tested strain Paer03. **c** Identical setup for the tested strain Paer09. **d** Identical setup for the tested strain Paer57. No phage plaques could be identified when spotting the initial phage solution (round 0) on Paer57. **e** Identical setup for the tested strain Paer60. **f** Identical setup for the tested strain Paer84. **g** Identical setup for the tested strain Paer85.

Source data are provided as a source data file.

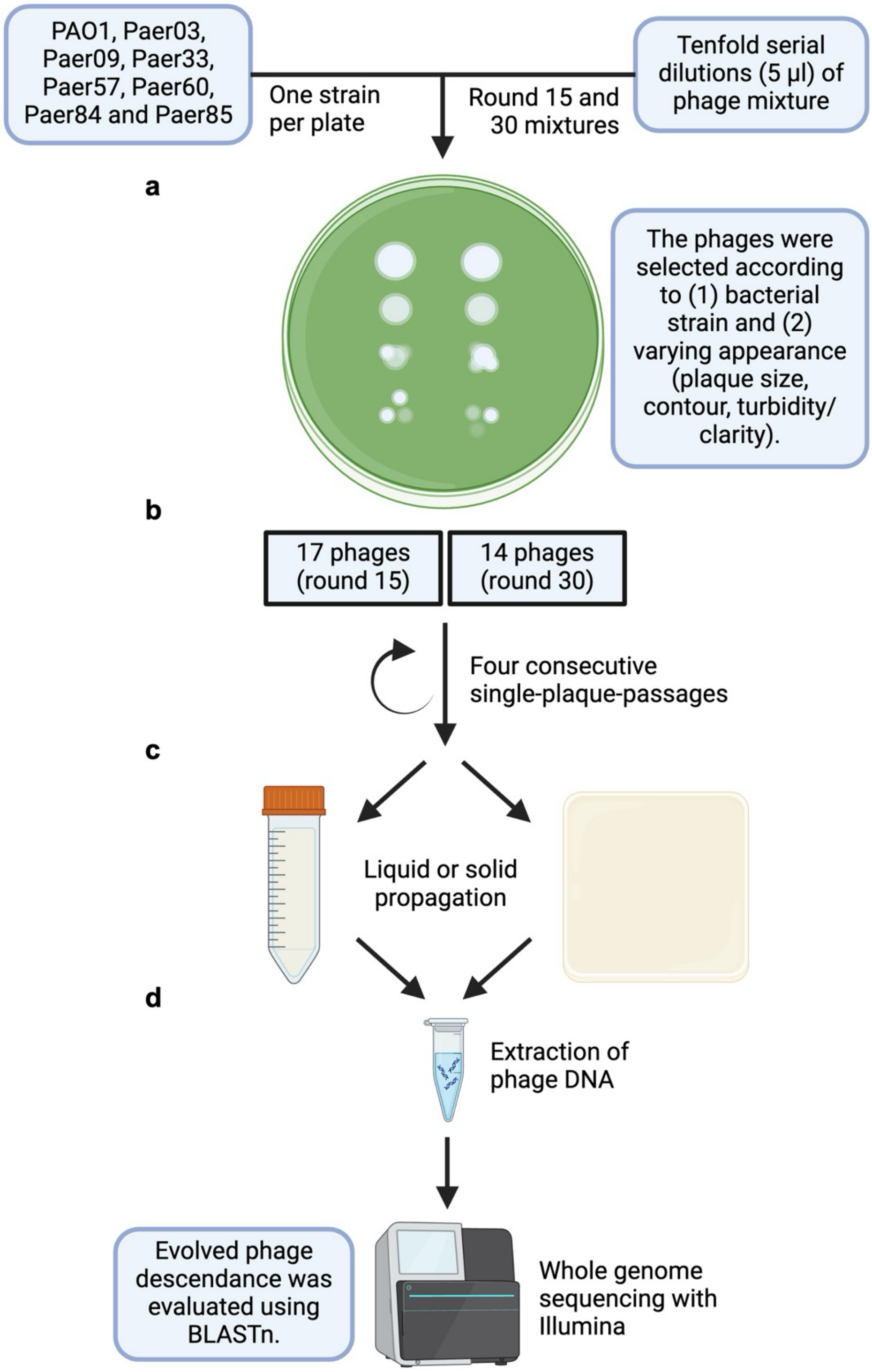

Fig. S3: Overview of the isolation of evolved bacteriophages after rounds 15 and 30.

a Tenfold serial dilutions of the bacteriophage (phage) mixtures after round 15 and 30 were individually spotted (5 µl) on soft agar overlays for each individual bacterial strain in the evolution assay (PAO1, Paer03, Paer09, Paer33, Paer57, Paer60, Paer84 and Paer85). **b** Based on the host strain and qualitative plaque assessment (plaques‘ size, contour, and turbidity/clarity) 17 evolved phages were picked from

round 15 spot assays and 14 from round 30. The phages were purified by four consecutive single-plaque-passages. **c** Each isolated phage was produced from a single plaque using either a liquid or solid propagation method. **d** Phage genomes were extracted, sequenced with Illumina, and assembled before the corresponding ancestral phage was identified using BLASTn.

Figure S3 was created with BioRender.com and released under a Creative Commons Attribution-NonCommercial-NoDerivs 4.0 International license (https://creativecommons.org/licenses/by-nc-nd/4.0/deed.en).

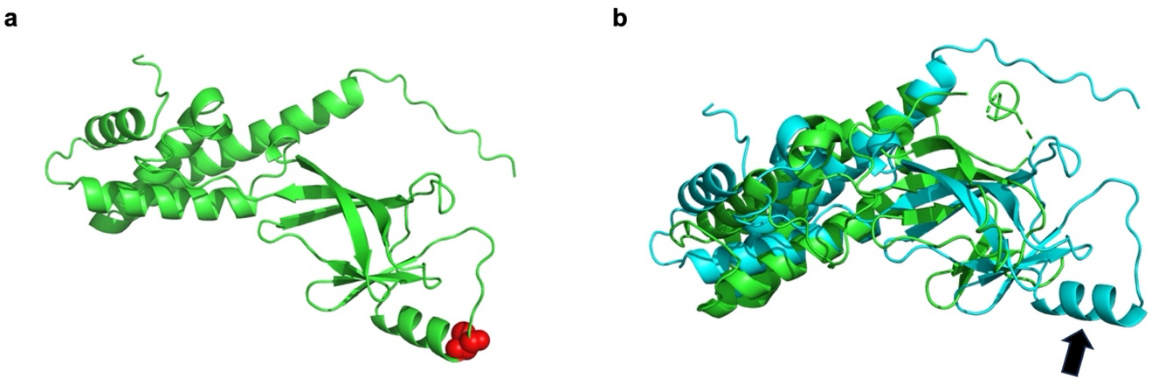

Fig. S4: Overview of the tertiary structure for FJK_gp62 and TTPA.

a Tertiary structure prediction of EPS depolymerase FJK_gp62. In red, the cysteine residue on position 98 is highlighted, which is mutated to the aromatic amino acid phenylalanine in the evolved phage. **b** Superimposition of EPS depolymerase FJK_gp62 (blue) and the tail tubular protein A (TTPA) of Klebsiella phage KP32 (green). Both structures contain a compact α-helical domain on the one side and β-strands and loops on the other side. These β-strands constitute two antiparallel β-sheets. FJK_gp62 contains an extra α-helix close to these β-sheets (arrow).

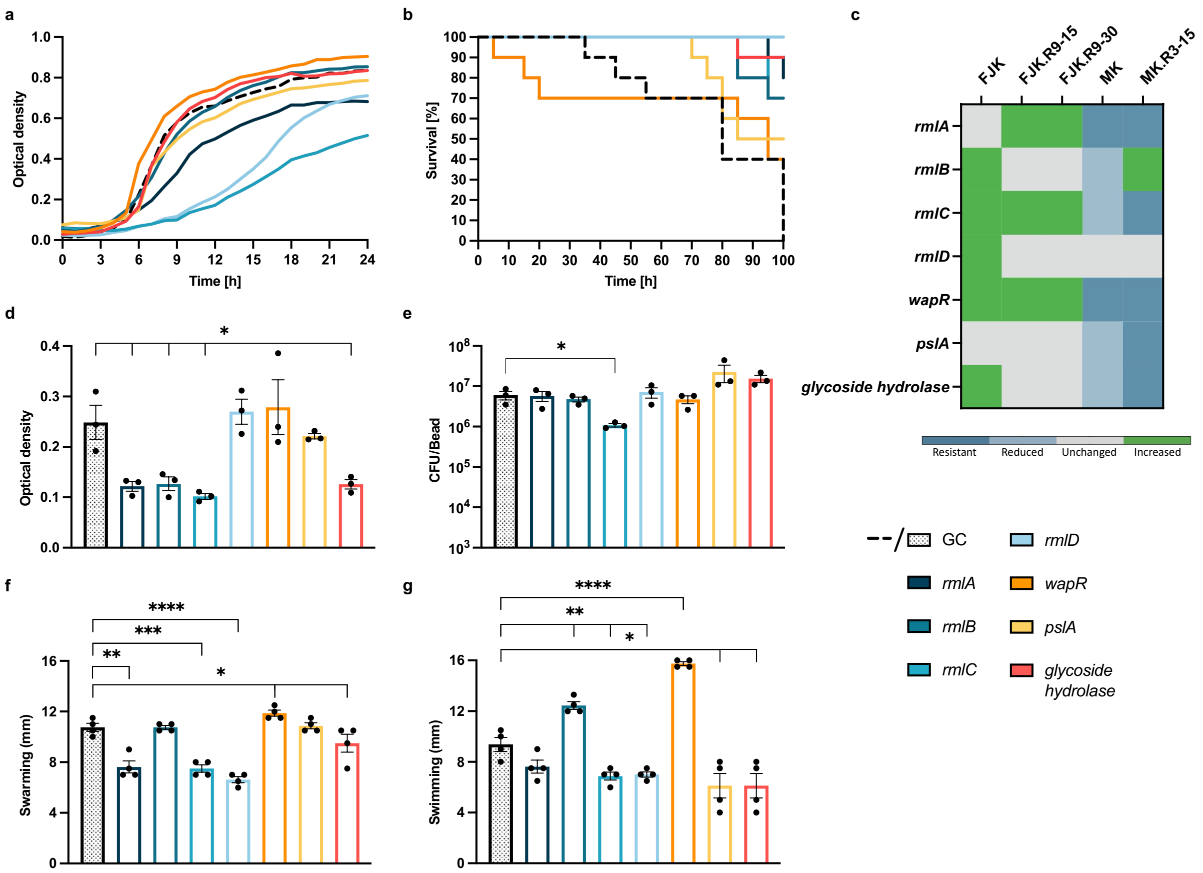

Fig. S5: Characterisation of phage treated Paer09 mutants.

a Optical density (OD_{600}) measurements of a 24 h incubation of the planktonic naive Paer09 strain (GC, growth control) alongside seven phage treated Paer09 mutants with one altered gene (*wapR*, frameshift; *pslA*, nonsense; *rmlA*, nonsense; *rmlB*, missense; *rmlC*, frameshift; *rmlD*, nonsense; *glycoside hydrolase*, frameshift) per mutant. Each line represents the average of three biological replicates with three underlying technical replicates corrected by the blank control (TSB). **b** Percentual survival of *Galleria mellonella* larvae after injection with 10 µl of bacteria (10^3 CFU/ml) (n=10 per strain; Paer09 and mutants stated above) or PBS (control; black line with no deaths; n=10) monitored for 100 h. **c** Efficacy of plating (EOP) of five phages (FJK, FJK.R9-15, FJK.R9-30, MK, and MK.R3-15) for seven representative Paer09 mutants (mutants stated above) complemented with their respective wild-type gene. The EOP is defined as the concentration ratio of a phage on the complemented mutant (numerator; plasmid with wild-type gene) and the non-complimented mutant (denominator; empty plasmid). An EOP above 10 was considered as "increased" efficiency (green), while an EOP of 0.1 to 10 was ranked as "unchanged" efficiency (grey). A "reduced" efficiency was defined as an EOP between 0.001 and 0.1 (light blue). When no individual plaques were visible or the EOP was equal to or under 0.001 the efficiency was

considered “greatly reduced” (dark blue). **d** Optical density (OD_{570}) measurements of crystal violet/ethanol solution obtained from stained bacterial microtiter plate biofilms (Paer09 and mutants stated above). **e** Bacterial load (CFU/bead) of 24 h pre-established bacterial biofilms (Paer09 and mutants stated above), determined by plating. **f** Swarming motility of each strain (Paer09 and mutants stated above) on TSA plates (0.5% w/v agar). **g** Swimming motility of each strain (Paer09 and mutants stated above) on TSA plates (0.3% w/v agar).

Unless specified otherwise, all experiments were performed in three biological replicates with either three (**a**, **d** and **e**) or no (**c**) technical replicates, except the motility which was determined in four biological replicates (**f** and **g**). The error bars represent the standard error of the mean. The statistical analysis was conducted by unpaired two-tailed Student’s *t* test and *p*-values were indicated by an asterisk (*, p=<0.05; **, p=<0.01; ***, p=<0.001; ****, p=<0.0001). Source data are provided as a source data file.
