# Supplementary material for: Targeting Pseudomonas aeruginosa biofilm with an evolutionary trained bacteriophage cocktail exploiting phage resistance trade-offs
Source: Nat Commun. 2024 Oct 3;15:8572. doi: 10.1038/s41467-024-52595-w (PMC11450229; doi:10.1038/s41467-024-52595-w)
Supplement: Supplementary file 3 — Reporting Summary [file 41467_2024_52595_MOESM3_ESM.pdf]

nature portfolio

Corresponding author(s): Andrej Trampuz

Last updated by author(s): Jul 31, 2024

Reporting Summary

Nature Portfolio wishes to improve the reproducibility of the work that we publish. This form provides structure for consistency and transparency in reporting. For further information on Nature Portfolio policies, see our Editorial Policies and the Editorial Policy Checklist.

Statistics

For all statistical analyses, confirm that the following items are present in the figure legend, table legend, main text, or Methods section.

n/a	Confirmed	
☐	☒	The exact sample size (*n*) for each experimental group/condition, given as a discrete number and unit of measurement
☐	☒	A statement on whether measurements were taken from distinct samples or whether the same sample was measured repeatedly
☐	☒	The statistical test(s) used AND whether they are one- or two-sided<br>*Only common tests should be described solely by name; describe more complex techniques in the Methods section.*
☒	☐	A description of all covariates tested
☒	☐	A description of any assumptions or corrections, such as tests of normality and adjustment for multiple comparisons
☐	☒	A full description of the statistical parameters including central tendency (e.g. means) or other basic estimates (e.g. regression coefficient) AND variation (e.g. standard deviation) or associated estimates of uncertainty (e.g. confidence intervals)
☐	☒	For null hypothesis testing, the test statistic (e.g. *F*, *t*, *r*) with confidence intervals, effect sizes, degrees of freedom and *P* value noted<br>*Give P values as exact values whenever suitable.*
☒	☐	For Bayesian analysis, information on the choice of priors and Markov chain Monte Carlo settings
☒	☐	For hierarchical and complex designs, identification of the appropriate level for tests and full reporting of outcomes
☐	☒	Estimates of effect sizes (e.g. Cohen's *d*, Pearson's *r*), indicating how they were calculated

Our web collection on statistics for biologists contains articles on many of the points above.

Software and code

Policy information about availability of computer code

Data collection	Excel; Guppy (v3.1.5); Unicycler hybrid assembly pipeline (v0.4.8.0); PATRIC genome assembly (v3.6.12); Shovill (v1.1.0)
Data analysis	Excel; BLASTn (v2.13.0); ImageJ (v1.54g); Bandage (v0.8.1); Prokka (v1.14.6); Roary (v3.13.0); RAxML (v8.2.4); iTOL (v6.5); Snippy (v4.6.0); VIRIDIC; MEGA11; RASTtk pipeline; HHpred; HHblits; HMMER (v.3.3); Artemis (v18.1.0); Easyfig (v2.2.2); ColabFold; DALI; PyMOL 2.5; BioRender; GraphPad Prism 9

For manuscripts utilizing custom algorithms or software that are central to the research but not yet described in published literature, software must be made available to editors and reviewers. We strongly encourage code deposition in a community repository (e.g. GitHub). See the Nature Portfolio guidelines for submitting code & software for further information.

Data

Policy information about availability of data

All manuscripts must include a data availability statement. This statement should provide the following information, where applicable:

- Accession codes, unique identifiers, or web links for publicly available datasets
- A description of any restrictions on data availability
- For clinical datasets or third party data, please ensure that the statement adheres to our policy

The genomic data generated and analysed during the current study is available under NCBI BioProject PRJNA906522 (https://www.ncbi.nlm.nih.gov/bioproject/

PRJNA906522.

Research involving human participants, their data, or biological material

Policy information about studies with human participants or human data. See also policy information about sex, gender (identity/presentation), and sexual orientation and race, ethnicity and racism.

Reporting on sex and gender	*Use the terms sex (biological attribute) and gender (shaped by social and cultural circumstances) carefully in order to avoid confusing both terms. Indicate if findings apply to only one sex or gender; describe whether sex and gender were considered in study design; whether sex and/or gender was determined based on self-reporting or assigned and methods used. Provide in the source data disaggregated sex and gender data, where this information has been collected, and if consent has been obtained for sharing of individual-level data; provide overall numbers in this Reporting Summary. Please state if this information has not been collected. Report sex- and gender-based analyses where performed, justify reasons for lack of sex- and gender-based analysis.*
Reporting on race, ethnicity, or other socially relevant groupings	*Please specify the socially constructed or socially relevant categorization variable(s) used in your manuscript and explain why they were used. Please note that such variables should not be used as proxies for other socially constructed/relevant variables (for example, race or ethnicity should not be used as a proxy for socioeconomic status). Provide clear definitions of the relevant terms used, how they were provided (by the participants/respondents, the researchers, or third parties), and the method(s) used to classify people into the different categories (e.g. self-report, census or administrative data, social media data, etc.) Please provide details about how you controlled for confounding variables in your analyses.*
Population characteristics	*Describe the covariate-relevant population characteristics of the human research participants (e.g. age, genotypic information, past and current diagnosis and treatment categories). If you filled out the behavioural & social sciences study design questions and have nothing to add here, write "See above."*
Recruitment	*Describe how participants were recruited. Outline any potential self-selection bias or other biases that may be present and how these are likely to impact results.*
Ethics oversight	*Identify the organization(s) that approved the study protocol.*

Note that full information on the approval of the study protocol must also be provided in the manuscript.

Field-specific reporting

Please select the one below that is the best fit for your research. If you are not sure, read the appropriate sections before making your selection.

☒ Life sciences ☐ Behavioural & social sciences ☐ Ecological, evolutionary & environmental sciences

For a reference copy of the document with all sections, see nature.com/documents/nr-reporting-summary-flat.pdf

Life sciences study design

All studies must disclose on these points even when the disclosure is negative.

Sample size	No statistical method was used to determine the sample size for our experiments in advance. Instead, sample sizes were selected based on inclusion criteria, previous similar studies in the field, the specific objectives of each experiment and practical considerations.<br><br>Sample sizes are listed below for all experiments performed in this study.<br><br>1. Evolution assay: Four ancestral phages and eight P. aeruginosa strains were selected based on the inclusion criteria.<br>2. Bacterial biofilm imaging: All eight P. aeruginosa strains from the evolution assay were imaged by scanning electron microscopy.<br>3. Phage imaging: All four ancestral phages from the evolution assay were imaged by transmission electron microscopy.<br>4. Evolved phage sequencing: Ten evolved phages, isolated after round 15 (n=5) and round 30 (n=5), were selected based on descent, as well as a distinct efficiency and host range.<br>5. Host range analysis: 80 P. aeruginosa strains including 79 clinical isolates (Belgium (n=41), Switzerland (n=17), Italy (n=6) and Germany (n=17)) and one laboratory reference strain (PAO1). All ancestral (n=4) and evolved (N=10) phages were tested.<br>6. Antimicrobial and antibiofilm analysis: Three ancestral phages and six evolved phages (round 15, n=3; round 30, n=3) were tested against two P. aeruginosa strains included in the evolution assay (Paer09, Paer57) and one strains not included (Per36). In addition, a phage cocktail (FJK.R9-30 and MK.R3-15) was tested against one P. aeruginosa strain (Paer09).<br>7. Phage treated Paer09: One P. aeruginosa strain (Paer09) was treated with one ancestral phage (FJK), two evolved phages (FJK.R9-15, FJK.R9-30) and a phage cocktail (FJK.R9-30 and MK.R3-15). Phage susceptibility to 66 isolates and a control (Paer09) was tested for five phages (FJK, FJK.R9-15, FJK.R9-30, MK, MK.R3-15).<br>8. : Characterization of phage treated Paer09 mutants: Seven bacterial isolates, each mutated in only one of the seven identified genes, were further characterized.
Data exclusions	No data was excluded from the analysis.
Replication	The reproducibility of our findings was successfully confirmed by conducting the experiments in replicates (biological and/or technical).<br><br>The number of replicates (biological and/or technical) are listed below for all experiments performed in this study.

1. Evolution assay: The assay was performed once (one biological replicate) as a proof of concept to demonstrate the feasability of our approach.
2. Bacterial biofilm imaging: From each bacteria included in the evolution one biological replicate was used for imaging.
3. Phage imaging: From each ancestral phage solution one biological replicate was used for imaging.
4. Evolved phage analysis: From each evolved phage solution one biological replicate was used for sequencing.
5. Host range analysis: The experiment was either conducted as two biological replicates with two technical replicates each (ancestral phages) or as three biological replicates (evolved phages)
6. Antimicrobial and antibiofilm analysis: The experiments were performed as two biological replicates with two technical replicates each.
7. Phage treated Paer09: This experiment was carried out in two biological replicates with four technical replicates (individual phages) or in eight biological replicates (cocktail).
8. : Characterization of phage treated Paer09 mutants (n=7): Growth curves were prepared in three biological replicates, with three technical replicates each. To test the virulence in G. mellonella ten larvae per strain were injected with bacteria and ten larvae were used as a control. For each strain the biofilm cell count determination was conducted in three biological replicates with two technical replicates each. The crystal violet staining was performed as three biological replicates with three technical replicates each. The swarming and swimming motility were performed in four biological replicates. Phage susceptibility was evaluated for each complemented and non-complemented mutant in biological triplicates.

Randomization: Randomization was not relevant for our study, as we worked with identical Pseudomonas aeruginosa strains for each experiment.

Blinding: Blinding was not relevant to our study, as we carried out an experimental microbiological study.

Reporting for specific materials, systems and methods

We require information from authors about some types of materials, experimental systems and methods used in many studies. Here, indicate whether each material, system or method listed is relevant to your study. If you are not sure if a list item applies to your research, read the appropriate section before selecting a response.

Materials & experimental systems

n/a	Involved in the study
☒	☐ Antibodies
☒	☐ Eukaryotic cell lines
☒	☐ Palaeontology and archaeology
☒	☐ Animals and other organisms
☒	☐ Clinical data
☒	☐ Dual use research of concern
☒	☐ Plants

Methods

n/a	Involved in the study
☒	☐ ChIP-seq
☒	☐ Flow cytometry
☒	☐ MRI-based neuroimaging
